# Suicidal behaviour and cognition: A systematic review with special focus on prefrontal deficits

**DOI:** 10.1192/j.eurpsy.2021.1558

**Published:** 2021-08-13

**Authors:** J. Fernández-Sevillano, A. González-Pinto, J. Rodríguez-Revuelta, S. Alberich-Mesa, L. González-Blanco, I. Zorrilla-Martínez, Á. Velasco, P. López-Pena, I. Abad-Acebedo, P.A. Saiz

**Affiliations:** 1 Medicine Department, Neuroscience, University of the Basque Country, LEIOA, Spain; 2 Bipolar Disorder Research Group, CIBERSAM, Madrid, Spain; 3 Department Of Psychiatry, Araba University Hospital, BIOARABA RESEARCH INSTITUTE Osakidetza Basque Health Service, Araba University Hospital, Vitoria-Gasteiz, Spain; 4 Medicine Departament, Neuroscience, University of the Basque Country, LEIOA, Spain; 5 Departametn Of Psychiatry, Araba University Hospital, BIOARABA RESEARCH INSTITUTE Osakidetza Basque Health Service, Araba University Hospital, Vitoria-Gasteiz, Spain; 6 Department Of Psychiatry, University of Oviedo, Oviedo, Spain; 7 Neuroscience And Sense Organs, ISPA HEALTH RESEARCH INSTITUTE OF THE PRINCIPALITY OF ASTURIAS, Oviedo, Spain; 8 Psychiatry, SESPA Mental Health Services of Principado de Asturias, OVIEDO, Spain; 9 Psychiatry, CIBERSAM, Madrid, Spain

**Keywords:** Suicide, cognition, Neuropsychological functioning

## Abstract

**Introduction:**

Suicidal behaviour and cognition: A systematic review with special focus on prefrontal deficits Introduction: Suicide is a major health concern worldwide, thus, identifying risk factors would enable a more comprehensive understanding and prevention of this behaviour. Neuropsychological alterations could lead to difficulties in interpreting and managing life events resulting in a higher risk of suicide.

**Objectives:**

Objective: Bibliographic review about the influence of neuropsychological deficits on suicidal behaviour.

**Methods:**

Method: A systematic literature search from 2000 to 2020 was performed in Medline (Pubmed), Web of Science, SciELO Citation Index, PsycInfo, PsycArticles and Cochrane Library databases regarding studies comparing cognition of attempters versus non-attempters that share same psychiatric diagnosis. Results: 1.885 patients diagnosed with an Affective Disorder (n = 1512) and Schizophrenia/ Schizoaffective Disorder (n = 373) were included.

**Results:**

In general comparison, attention was found to be clearly dysfunctional. Regarding diagnosis, patients with Schizophrenia and previous history of suicidal behaviour showed a poorer performance in executive function. Patients with current symptoms of an Affective Disorder and a previous history of suicidal attempt had poorer performance in attention and executive function. Similarly, euthymic affective patients with history of suicidal behaviour had worse decision-making, attention and executive function performance compared to euthymic non-attempters.

**Conclusions:**

Patients who have attempted suicide have a poorer neuropsychological functioning than non-attempters with a similar psychiatric disorder in attention and executive function. These alterations increase vulnerability for suicide.

**Conflict of interest:**

Jessica Fernández-Sevillano is beneficiary of the Pre-PhD Training Programme of the Basque Government. Dr. Gonzalez-Pinto has received grants and served as consultant, advisor or CME speaker for the following entities: Almirall, AstraZeneca, Bristol-Myers

